# The Charges against Hinckley Isolation Hospital

**Published:** 1912-05-18

**Authors:** Myra Forsyth

**Affiliations:** Matron. Isolation Hospital


*To the Editor of* The Hospital.

### The Charges against Hinckley Isolation Hospital.

*To the Editor of* The Hospital.

Sir,—Since you are so generous as to reserve your comments pending the inquiry into the "serious charges" against the staff of this hospital, a few particulars concerning the said hospital may prove both interesting and enlightening. There are two blocks, each containing 12 beds; they are of the galvanised nature beloved of provincial committees. They are in a deplorable, tumble-down, and dilapidated condition, having been in existence on present site since 1896, prior to which they were used for smallpox at a place about three-and-a-half miles from here. I may mention that both rain and snow penetrate.

Prior to last August each block was served by one closet-arrangement with a bucket, the condition of which must have been seen to be believed. In August the committee consented to erect two additional conveniences of the same nature on account of our having at that time between 40 and 58 patients, a number of whom were adults. Extra beds were put up in the wards, and two large tents were erected. I was not restricted as to staff, but having only four small bedrooms in the administration block (a permanent building erected in 1907-1908), and having to provide accommodation for the domestic staff, you will understand I was unable to be very lavish as regards nurses. As a matter of fact, I managed by utilising outside sheds to accommodate four, in addition to which I had one general servant and one wardmaid, and two daily charwomen who did the washing; so I can hardly be said to have been overstaffed. The cooking was superintended by me personally, assisted by the "general" alluded to.

The tents were taken down owing to the bad weather on October 1, and the patients were transferred to the two blocks, making at that time 42 in all, which remained the average number until after Christmas, the nursing staff being reduced to three, at which number it remained until the beginning of December, when it was reduced to two. From October 1 until December 27 I was also in charge of, and responsible for, the nursing of patients in their homes in a very large district, both urban and rural. I had two nurses at work on this, and their work, etc., was organised and superintended by me.

I may mention that during the whole time the epidemic lasted I had a great difficulty in keeping nurses, the conditions under which they had to work being the contrary of attractive, although personally I tried to make them as comfortable as possible.

With regard to the complaint made to the committee (made, by the way, some six or seven weeks after the children had been discharged) my reply *should* have been sufficient, and I have practically nothing to add to it except, that on Monday* I shall produce report-books, ambulance books, and charts to prove what I have said; also the two nurses who were on duty in the wards.

With regard to the allegation concerning the dying child, I enclose a time-table for the date alluded to. I intend, unless this charge is unreservedly and absolutely withdrawn, to instruct my solicitor to issue a writ for slander and claim damages from the parties concerned. The dying child's father had free access to the ward during the day and evening, and paid a last visit at 8.45 P.M.

The children responsible for these startling statements were, I have since been informed, responsible for statements equally startling concerning their home and parents, which they made with great freedom to the other patients in the ward.

I am astonished that a professional paper like The Hospital should stoop to circulate or give any publicity to such statements until they had been proved to be true. One's professional reputation is not likely to be enhanced by such publicity in circumstances like these, and surely we suffer enough from sensational reporting in the local press!

Your way of quoting, too, is misleading. From reading your paragraph one might imagine that I meant that fully 90 per cent. of the patients here are in a verminous condition. What I said was that they *were admitted* in a verminous condition. I certainly never meant anyone to suppose they were allowed to remain like that. In conclusion, I may state that all the nurses behaved well under the most trying conditions, and the mortality during the epidemic was less than 2 per cent., although the type of scarlet fever was in most cases severe. If you will remember that our hospital is supposed to accommodate 24 patients at its utmost capacity, and that from June 1911 to April 1912 I dealt with 262 patients, I think you must acknowledge a death-rate of less than 2 per cent. is something to be proud of taking all the disadvantages into consideration. In all the years the hospital has been open the number of patients for the year rarely exceeded 57. You can use your own discretion as to the use you make of this letter.—Yours faithfully,

Myra Forsyth, Matron.

Isolation Hospital, May 11.

* [This letter was written before the meeting last Monday, which is reported in our Notes this week.—Ed., The Hospital.]

